# AI for glaucoma, Are we reporting well? a systematic literature review of DECIDE-AI checklist adherence

**DOI:** 10.1038/s41433-025-03678-5

**Published:** 2025-02-18

**Authors:** Benedict Leonard-Hawkhead, Bethany E. Higgins, David Wright, Augusto Azuara-Blanco

**Affiliations:** 1 Centre for Public Health Queens University Belfast, Belfast, UK; 2 Optometry and Visual Sciences, School of Health Sciences, City, University of London, London, UK

**Keywords:** Medical research, Education

## Abstract

**Background/Objectives:**

This systematic literature review examines the quality of early clinical evaluation of artificial intelligence (AI) decision support systems (DSS) reported in glaucoma care. Artificial Intelligence applications within glaucoma care are increasing within the literature. For such DSS, there needs to be standardised reporting to enable faster clinical adaptation. In May 2022, a checklist to facilitate reporting of early AI studies (DECIDE-AI) was published and adopted by the EQUATOR network.

**Methods:**

The Cochrane Library, Embase, Ovid MEDLINE, PubMed, SCOPUS, and Web of Science Core Collection were searched for studies published between January 2020 and May 2023 that reported clinical evaluation of DSS for the diagnosis of glaucoma or for identifying the progression of glaucoma driven by AI. PRISMA guidelines were followed (PROSPERO registration: CRD42023431343). Study details were extracted and were reviewed against the DECIDE-AI checklist. The AI-Specific Score, Generic-Item Score, and DECIDE-AI Score were generated.

**Results:**

A total of 1,552 records were screened, with 19 studies included within the review. All studies discussed an early clinical evaluation of AI use within glaucoma care, as defined by the a priori study protocol. Overall, the DECIDE-AI adherence score was low, with authors under reporting the AI specific items (30.3%), whilst adhering well to the generic reporting items (84.7%).

**Conclusion:**

Overall, reporting of important aspects of AI studies was suboptimal. Encouraging editors and authors to incorporate the checklist will enhance standardised reporting, bolstering the evidence base for integrating AI DSS into glaucoma care workflows, thus help improving patient care and outcomes.

## Introduction

Glaucoma is an optic neuropathy that manifests with peripheral vision loss. It is a progressive and irreversible condition with a poorly understood aetiology. Clinically, glaucoma can pose a challenge to diagnose [1], therefore, clinical examination by senior ophthalmologists, visual field (VF) measurements, optical coherence tomography (OCT), along with other clinical tests are all used to determine if a patient has glaucoma [2]. Glaucoma comprises of a heterogeneous group of disorders, that are broadly categorised as open angle glaucoma (OAG) and angle closure glaucoma (ACG), defined by the irideocorneal angle [3]. The current prevalence estimate of glaucoma worldwide is 76 million, and this statistic is estimated to increase to 111.8 million by 2040 [4]. With 11% of world blindness in adults over 50 years being attributed to glaucoma in 2020, glaucoma is a leading public health concern and burden for healthcare systems worldwide [5]. Within the United Kingdom (UK), glaucoma care accounts for 20% of out-patient hospital eye services workload [6], and represents a significant financial burden to health care providers.

Within many disciplines of medicine, there has been a stark increase within the body of literature citing artificial intelligence (AI) applications to assist medical practitioners [7]. These applications show promise that could lead to physicians having more time to focus on high-yield activities such as surgical waiting lists, though despite the promise of these decision support systems (DSS) they must undergo the same scrutiny as any drug, medical device, or surgical innovation. The development pathways for such interventions are unclear as DSS lack compelling definitions of study stage that exist for drug trials and surgical innovations [8]. In order for the body of literature to be put into effect within the practice of medicine, DSS using AI must be supported with systematic reporting in order to build a sound, robust and comprehensive body of evidence. The DECIDE-AI guideline was published in May 2022 to give authors a standardised template to use when reporting early-stage clinical evaluation of such systems [8].

The DECIDE-AI guideline is a “stage specific reporting guideline for the early, small scale and live clinical evaluation of DSS based on AI” [8]. It was developed under a consensus process involving 20 stakeholder groups across 18 countries with 151 experts involved in the process. The checklist provides a framework of minimum reporting standards and is comprised of 27 items. The DECIDE-AI checklist is made of two parts: 17 AI specific reporting items, and 10 generic reporting items. Such checklists aid the synthesis of standardised evidence, to improve appraisal and replicability of the study findings.

In this systematic review we aim to evaluate the current reporting of AI DSS in glaucoma care. The review specifically examines the body of literature for AI DSS used to diagnose glaucoma and/or its progression to assess how well authors report their findings in the early clinical evaluation.

## Methods

### Literature search strategy

The study protocol was registered on PROSPERO (ID: CRD42023431343) and the systematic review followed the Preferred Reporting Items for Systematic Reviews and Meta-Analyses (PRISMA) guidelines [9]. A list of the search terms are provided within the supplementary materials (Fig. S1), which were used to systematically search: The Cochrane Library, Embase, Ovid MEDLINE, PubMed, SCOPUS, and Web of Science Core Collection. Databases were searched between 01/01/2020 – 25/05/2023. The search strategy used key terms and subject headings relating to the three themes of this review: (1) AI, (2) Glaucoma, and (3) Detection/Progression. Identified records were exported into Rayyan where automatic duplication of records were reviewed [10]. EndNote 20 was used to manage selected articles [11].

### Study selection

The articles identified from the bibliographic search were de-duplicated using Rayyan software and subsequently underwent screening by title and abstract, completed independently by two authors (BLH and BEH), following the predefined criteria. To be eligible for inclusion, studies had to be: (1) published in the English language; (2) dated from January 2020 to current; (3) include glaucoma patients; and (4) include use of AI, machine learning, or deep learning to diagnosis or predict progression. Studies were excluded if they were review articles, letters to the editor, published protocols, conference abstracts, or non-human studies. Papers that were unclear on these criteria were brought to full-text screening.

Articles that passed screening by title and abstract were brought to full text review. Two authors independently screened the articles by full text to determine their eligibility according to the following criteria: (1) use of any AI to diagnosis or predict glaucoma, (2) early clinical evaluation (informed consent received), and (3) full text published in English. Conflicts were discussed and resolved through consensus. If a consensus could not be agreed the article was passed to a third author (AAB) for arbitration.

### Data collection and synthesis

The articles identified for inclusion underwent data extraction by one investigator (BLH) and data was inputted into a data synthesis table. Information extracted from each article included: Title, Publication Date, Author, Journal, Input data, Aim, Method (Deep Learning/Machine Learning), and review against the DECIDE-AI Checklist. A meta-analysis was not appropriate given the diversity of the publications and aim to review adherence to the DECIDE-AI checklist.

### DECIDE-AI checklist

The DECIDE-AI guideline was produced under an international consensus process to aid better reporting of early clinical evaluation of DSS using AI [8]. The review against the DECIDE-AI included both the 17 AI specific reporting items and the 10 generic reporting items. The AI specific reporting items include a further 11 sub-sections, thus the highest score for the purposes of this review was 28.

The DECIDE-AI checklist was used in this review to assess how well authors adhered to the guidance; using a binary system in which reported was denoted by a ‘1’ and ‘0’ represented not reported. Though the checklist provides 27 reporting items, it also includes recommendations. For example, AI specific item two has two subparts, making an enquiry into both the targeted medical condition and the intended user. These subparts were also assessed using the same binary scoring described above and therefore, if an author reports all items on the checklist the paper could ‘score’ a maximum of 38; 28 points for the AI and 10 for the Generic items.

Reporting item 13, safety and errors has two parts but has added complexity. Part a assesses the reporting of safety and errors and gives four further domains to consider, for this systematic review, a paper would receive a point for the subpart if it reported any of the further domains. Part b assesses risk to patient safety and does not give domains to consider, therefore a paper would receive ‘1’ if it considered aspects of patient safety.

The overall score, the AI specific score and generic score were collated and recorded within a data synthesis table. In addition to the raw score, means of the score are reported to convey average adherence to the DECIDE-AI checklist, with median reported to account for potential skewness or outliers, providing a more robust measure of central tendency. The DECIDE-AI checklist is provided below in Table 1 [8].

**Table 1:** DECIDE-AI reporting item checklist.

Item n0	Theme	Recommendation	Reported on page
1 - 17	AI-specific reporting items		
I - X	Generic Reporting Items		
Title and abstract			
1	Title	Identify the study as early clinical evaluation of a decision support system based on AI or machine learning, specifying the problem addressed.	
I	Abstract	Provide a structured summary of the study. Consider including: intended use of the AI system, type of underlying algorithm, study setting, number of patients and users included, primary and secondary outcomes, key safety endpoints, human factors evaluated, main results, conclusions.	
Introduction			
2	Intended use	a) Describe the targeted medical condition(s) and problem(s), including the current standard practice, and the intended patient population(s).	
		b) Describe the intended users of the AI system, its planned integration in the care pathway, and the potential impact, including patient outcomes, it is intended to have.	
II	Objectives	State the study objectives.	
Methods			
III	Research governance	Provide a reference to any study protocol, study registration number, and ethics approval.	
3	Participants	a) Describe how patients were recruited, stating the inclusion and exclusion criteria at both patient and data level, and how the number of recruited patients was decided.	
		b) Describe how users were recruited, stating the inclusion and exclusion criteria, and how the intended number of recruited users was decided.	
		c) Describe steps taken to familiarise the users with the AI system, including any training received prior to the study.	
4	AI system	a) Briefly describe the AI system, specifying its version and type of underlying algorithm used. Describe, or provide a direct reference to, the characteristics of the patient population on which the algorithm was trained and its performance in preclinical development/validation studies.	
		b) Identify the data used as inputs. Describe how the data were acquired, the process needed to enter the input data, the pre-processing applied, and how missing/low-quality data were handled.	
		c) Describe the AI system outputs and how they were presented to the users (an image may be useful).	
5	Implementation	a) Describe the settings in which the AI system was evaluated.	
		b) Describe the clinical workflow/care pathway in which the AI system was evaluated, the timing of its use, and how the final supported decision was reached and by whom.	
IV	Outcomes	Specify the primary and secondary outcomes measured.	
6	Safety and errors	a) Provide a description of how significant errors/malfunctions were defined and identified.	
		b) Describe how any risks to patient safety or instances of harm were identified, analysed, and minimised.	
7	Human factors	Describe the human factors tools, methods or frameworks used, the use cases considered, and the users involved.	
V	Analysis	Describe the statistical methods by which the primary and secondary outcomes were analysed, as well as any prespecified additional analyses, including subgroup analyses and their rationale.	
8	Ethics	Describe whether specific methodologies were utilised to fulfil an ethics- related goal (such as algorithmic fairness) and their rationale.	
VI	Patient involvement	State how patients were involved in any aspect of: the development of the research question, the study design, and the conduct of the study.	
Result			
9	Participants	a) Describe the baseline characteristics of the patients included in the study, and report on input data missingness.	
		b) Describe the baseline characteristics of the users included in the study.	
10	Implementation	a) Report on the user exposure to the AI system, on the number of instances the AI system was used, and on the users’ adherence to the intended implementation.	
		b) Report any significant changes to the clinical workflow or care pathway caused by the AI system.	
VII	Main results	Report on the prespecified outcomes, including outcomes for any comparison group if applicable.	
VIII	Subgroups analysis	Report on the differences in the main outcomes according to the prespecified subgroups.	
11	Modifications	Report any changes made to the AI system or its hardware platform during the study. Report the timing of these modifications, the rationale for each, and any changes in outcomes observed after each of them.	
12	Human-computer agreement	Report on the user agreement with the AI system. Describe any instances of and reasons for user variation from the AI system’s recommendations and, if applicable, users changing their mind based on the AI system’s recommendations.	
13	Safety and errors	a) List any significant errors/malfunctions related to: AI system recommendations, supporting software/hardware, or users. Include details of: (i) rate of occurrence, (ii) apparent causes, (iii) whether they could be corrected, and (iv) any significant potential impacts on patient care.	
		b) Report on any risks to patient safety or observed instances of harm (including indirect harm) identified during the study.	
14	Human factors	a) Report on the usability evaluation, according to recognised standards or frameworks.	
		b) Report on the user learning curves evaluation.	
Discussion			
15	Support for intended use	Discuss whether the results obtained support the intended use of the AI system in clinical settings.	
16	Safety and errors	Discuss what the results indicate about the safety profile of the AI system. Discuss any observed errors/malfunctions and instances of harm, their implications for patient care, and whether/how they can be mitigated.	
IX	Strengths and limitations	Discuss the strengths and limitations of the study.	
Statements			
17	Data availability	Disclose if and how data and relevant code are available.	
X	Conflicts of interest	Disclose any relevant conflicts of interest, including the source of funding for the study, the role of funders, any other roles played by commercial companies, and personal conflicts of interest for each author.	

## Results

### Search results

**Fig. 1:**
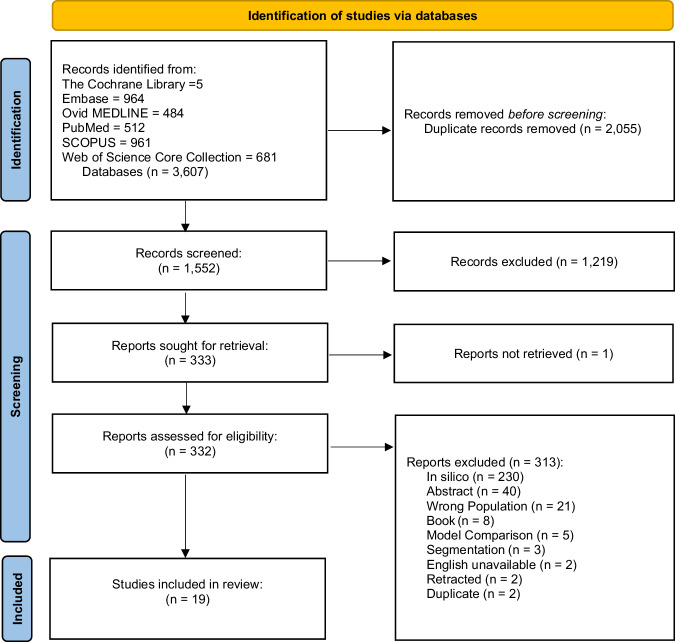
PRISMA flow diagram showing the selection of relevant articles identified from the literature search. PRISMA flow diagram of the selection of studies for inclusion in the systematic review.

The study selection process is outlined in the PRISMA flow diagram (Fig. 1). The predefined bibliographic search strategy yielded 3,607 articles, of which 2,055 were removed after duplicate detection using Rayyan. Title and abstract review removed a further 1,219 papers, leaving 333 papers to be reviewed by full text. Only one article, by Touahri et al., was not accessible via our institution or network [12]. The primary reason for exclusion during secondary screening was articles reporting within the in silico phase. From full text review, 19 papers met the study criteria and were included for data extraction.

### Study characteristics

Data from the selected studies has been tabulated and presented in Table 2. All studies selected for data extraction made use of AI and had received informed consent from the patient.

**Table 2:** Extracted data.

Title	Publication Date (MM/YYYY)	Lead Author	Journal	Input	Aim	Method (DL/ML)	AI-Specific Items	Generic Items	DECIDE-AI Score
A cascade eye diseases screening system with interpretability and expandability in ultra-wide field fundus images: A multicentre diagnostic accuracy study [16]	09/2022	Cao et a.l	eClinical Medicine	Ultra-wide fundus photographs	Identification of Disease	Deep Learning	12	9	21
Deep Learning Approach for Classification of the Primary Angle-closure Disease Spectrum Based on Anterior Segment Optical Coherence Tomography [31]	06/2023	Eslami et al.	Journal of Glaucoma	OCT	Identification of Subtypes	Deep Learning	8	9	17
A group of novel indexes of optical coherence tomography for computer-aided diagnosis of glaucoma [29]	07/2021	Xu et al.	Journal of Nonlinear and Convex Analysis	OCT	Identification of Disease	Machine Learning	7	9	16
A Multicenter Clinical Study of the Automated Fundus Screening Algorithm [21]	07/2022	Li et al.	Translational Vision Science & Technology	Fundus Images	Identification of Disease	Deep Learning	13	9	22
A Novel Hierarchical Deep Learning Framework for Diagnosing Multiple Visual Impairment Diseases in the Clinical Environment [17]	06/2021	Hong et al.	Frontiers in Medicine	Corneal Surface Images Fundus Images	Identification of Disease	Deep Learning	10	8	18
Agreement of a Novel Artificial Intelligence Software with Optical Coherence Tomography and Manual Grading of the Optic Disc in Glaucoma [26]	04/2023	Shroff et al.	Journal of Glaucoma	Fundus Images	Identification of Disease	Deep Learning	9	9	18
Artificial Intelligence for Screening of Multiple Retinal and Optic Nerve Diseases [20]	05/2022	Dong et al.	JAMA Network Open Ophthalmology	Fundus Images	Identification of Disease	Deep Learning	11	9	20
Comparison of different machine learning classifiers for glaucoma diagnosis based on Spectralis OCT [14]	09/2021	Wu et al.	Diagnostics	OCT	Identification of Disease	Machine Learning	7	9	16
Describing the Structural Phenotype of the Glaucomatous Optic Nerve Head Using Artificial Intelligence [25]	03/2022	Panda et al.	American Journal of Ophthalmology	OCT	Identification of Disease	Deep Learning	5	7	12
Gaze Exploration Index (GE i)-Explainable Detection Model for Glaucoma [18]	07/2022	Krishnan et al.	IEEE Access	Eye Movement Tracking	Identification of Disease	Deep Learning	8	7	15
Glaucoma Detection Using Support Vector Machine Method Based on Spectralis OCT [15]	02/2022	Wu et al.	Diagnostics	OCT and Clinical Parameters	Identification of Disease	Machine Learning	5	8	13
GlauCUTU: Time Until Perceived Virtual Reality Perimetry with Humphrey Field Analyzer Prediction-Based Artificial Intelligence [19]	03/2022	Kunumpol et al.	IEEE Access	VR Headset	Identification of Disease	Deep Learning	9	9	18
Medical Application of Geometric Deep Learning for the Diagnosis of Glaucoma [27]	02/2023	Thiéry et al.	Translational Vision Science & Technology	OCT	Identification of Disease	Deep Learning	5	9	14
Multimodal Machine Learning Using Visual Fields and Peripapillary Circular OCT Scans in Detection of Glaucomatous Optic Neuropathy [28]	02/2022	Xiong et al.	Ophthalmology	OCT and VF	Identification of Disease	Deep Learning	11	9	20
Redundancy reduced depthwise separable convolution for glaucoma classification using OCT images [13]	09/2021	Prabhakaran et al.	Biomedical Signal Processing and Control	OCT	Identification of Disease	Deep Learning	6	5	11
Risk of Normal Tension Glaucoma Progression from Automated Baseline Retinal-Vessel Caliber Analysis: A Prospective Cohort Study [23]	10/2022	Lin et al.	American Journal of Ophthalmology	Fundus Images	Identification of Disease	Deep Learning	6	9	15
Time-Frequency Analysis of ERG with Discrete Wavelet Transform and Matching Pursuits for Glaucoma [24]	10/2022	Sarossy et al.	Translational Vision Science & Technology	Electroretinography	Identification of Disease	Machine Learning	6	9	15
Use of Multimodal Dataset in AI for Detecting Glaucoma Based on Fundus Photographs Assessed with OCT: Focus Group Study on High Prevalence of Myopia [22]	11/2022	Lim et al.	BMC Medical Imaging	Fundus Images and OCT-A	Identification of Disease	Machine Learning and Deep Learning	9	9	18
Development and clinical deployment of a smartphone-based visual eld deep learning system for glaucoma detection [30]	09/2020	Li et al.	npj Digital Medicine	VF	Identification of Disease	Deep Learning	14	9	23

*OCT* optical coherence tomography, *VF* Visual Field.

Generally from the selected articles, authors aimed to diagnose glaucoma using AI [13–30], while one study aimed to identify subtypes of glaucoma [31]. No papers attempted to predict the progression of glaucoma. The selected studies primarily came from the medical literature (78.9%) [30, 31], while 10.5% came from engineering [18, 19], 5.3% from mathematical [29], and 5.3% from cross-disciplinary [13] journals. On average, papers within the medical literature performed better overall, but papers within the engineering literature performed better for the AI-specific items, while mathematical journals adhered best to the generic reporting items, see Table 3.

**Table 3:** Mean scores of total points across the different disciplines.

	AI-Specific Reporting Item Score (Max = 28)	Generic Reporting Item Score (Max = 10)	DECIDE-AI Score (Max = 38)
	Mean		
**Cross-disciplinary** (*n* = **1)**	6.0	5.0	11.0
**Engineering** (*n* = **2)**	8.5	8.0	16.5
**Mathematical** (*n* = **1)**	7.0	9.0	16.0
**Medical** (*n* = **15)**	8.7	8.7	17.5

Authors predominantly used deep learning (73.7%) [13, 23, 30, 31] to diagnose glaucoma though 21.1% of the papers used machine learning [14, 15, 24, 29] to identify glaucoma and one paper(5.3%) [22] used a mixture of both deep learning and machine learning to achieve the aim, Supplementary Materials S2: Distribution of AI Method used.

Authors used many different inputs, such as ultra-wide fundus photograph, OCT, fundus image, eye movement tracking, VR headset, visual field (VF) and electroretinography. The most commonly used imaging modality used was OCT with 31.6% of selected studies using it, the distributions of the other inputs can be seen in Supplementary Materials S3: Distribution of Data inputs.

### DECIDE-AI checklist

**Fig. 2:**
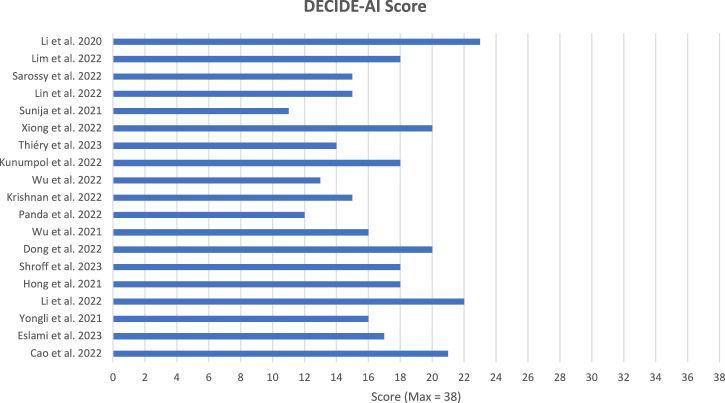
Bar chart showing the overall DECIDE-AI Score for included articles (Max score = 38). DECIDE-AI Score (Note: Max Score was 38).

No author referenced the DECIDE-AI guidance within their publication. Generally, adherence to the checklist was low, with an overall mean adherence of 44.6%, the median score was 17. The maximum score possible was 38 points. Figure 2 shows the total DECIDE-AI score for each paper out of a possible 38 points. The highest scoring paper was by Li et al. [30]. achieving 23 points out of 38 and the lowest score of 11 for Sunija et al. [13].

### Generic reporting items

**Fig. 3:**
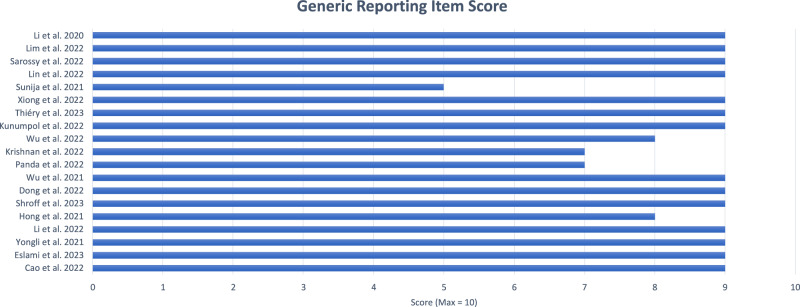
Bar chart showing the overall Generic Reporting Item Score for included articles (Max score = 10). Generic Reporting Item Score (Note: Max score was 10).

Overall, authors reported most of the generic reporting items, with a mean adherence of 84.7%. The highest possible score for this section was 10, the median score was 9. Of the 19 papers included within this review, 14 papers [14, 16, 26–31] scored 90%, 2 papers [15, 17] scored 80%, 2 papers [18, 25] scored 70%, and 1 paper [13] scored 50%, individual scores can be reviewed in Fig. 3.

**Fig. 4:**
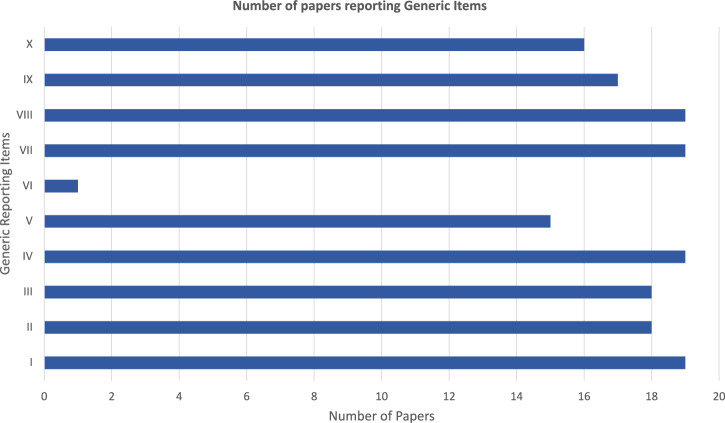
Bar chart showing the number of articles reporting each of the Generic Reporting Items. Total number of studies reporting each generic reporting item (Note: Max Score = 10).

More broadly, across the 19 included studies, all studies adhered to generic reporting items I (abstract), IV (outcomes), VII (main results) and VIII (subgroup analysis). Generic reporting item VI, patient involvement was least reported with only one paper referencing it. Figure 4 shows the respective overall score for each reporting item.

### AI-specific reporting items

**Fig. 5:**
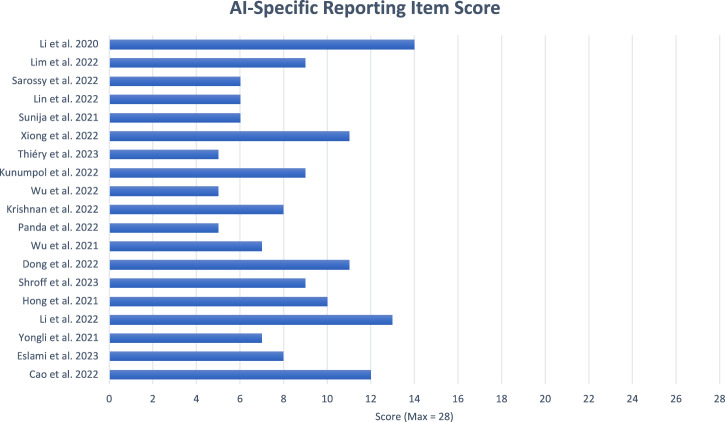
Bar chart showing the overall AI-Specific Score for included articles (Max Score = 28). AI-Specific Reporting Item Score (Note: Max Score = 28).

Across the 19 included papers, the mean adherence to the AI specific reporting items was low at 30.3%. The highest possible score for this section was 28, the median score was 8 and the highest score was 14, published by Li et al [30]. The lowest, and more prevalent score was 5, with three papers [15, 25, 27] attaining this result. Figure 5 shows the individual study score.

**Fig. 6:**
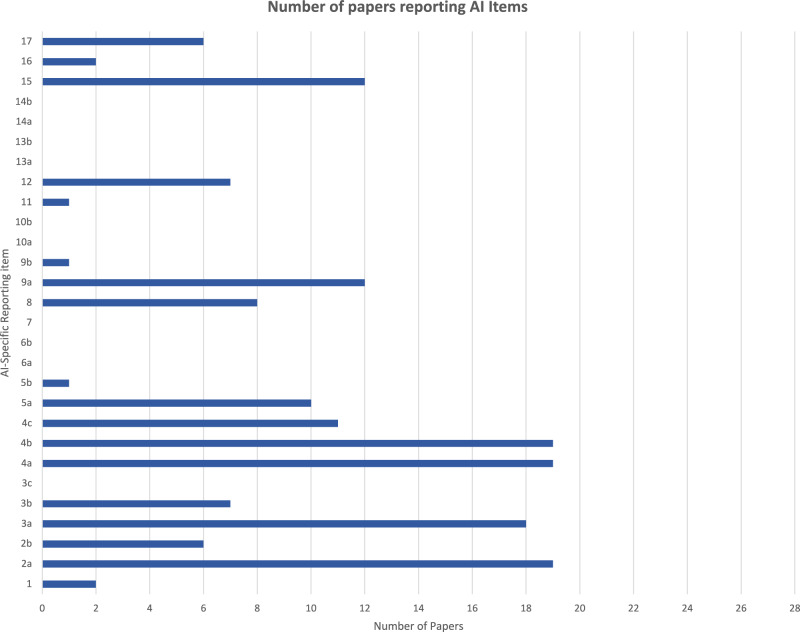
Bar chart showing the number of articles reporting each of the AI Specific Reporting Items. Total number of studies reporting each AI-specific reporting item (Note: Max Score 28).

All 19 studies adhered to the AI specific reporting items 2a (targeted medical condition), 4a (describe AI system) and 4b (describe data input), while items 3c (participants), 6a, 6b (safety and errors), 7 (human factors), 10a, 10b (implementation), 13a (safety and errors), 14a and 14b (human factors) were not reported at all. Figure 6 shows how many papers reported each of the AI specific items.

## Discussion

This systematic review aimed to determine how well the current literature citing early clinical evaluation of AI DSS to detect glaucoma and/or predict its progression is reported. The DECIDE-AI reporting guideline was used to generate a score of adherence and therefore assess how well each of the included papers reported according to the guideline. The guideline aims to standardise the reporting of early clinical evaluation of DSS and help provide clarity on this stage of evaluation. The definition of ‘early live clinical evaluation’ for AI studies is ambiguous and less well defined than the stages of drug trials and surgical innovations. The DECIDE-AI reporting guideline was first published in May 2022 [8]. Many of the studies included within this review were published in 2021, the year preceding the DECIDE-AI publication.

Artificial intelligence is a popular topic across all areas of the literature, with applications spanning from agriculture to medicine. By nature, AI is a cross-disciplinary field based within computer science, which can give rise to complexities when reporting AI methods within fields where there are already well-established research and reporting methods. Papers within this review were predominantly from the medical literature (15), with two from the discipline of engineering, and one from mathematics. One of the included papers were published within a cross-disciplinary journal. Those included from the medical literature scored the highest with an overall DECIDE-AI score of 17.5/38 (median = 18.0) with the cross-disciplinary paper scoring the lowest mean score of 11/38. For the AI specific reporting items, papers from medical journals scored the highest with a mean score of 8.7/28 (median = 9.0), while the paper published in the mathematical journal scored the highest (9/10) for the generic reporting items. A potential reason for the overall score being highest within the medical literature could be due to the DECIDE-AI checklist being designed for early clinical evaluations, and authors outside this field may not be accustomed to how evidence is reported in the medical field. Publications within other disciplines are reported in a different manner to that seen within medical journals.

Overall, adherence to the DECIDE-AI checklist (maximum =38) was low, brought around by low reporting of the AI specific items (maximum = 28), with a mean score of 30.3% (median = 8.0). The generic reporting items (maximum = 10) were reported well with an 84.7% mean adherence to the checklist (median = 9). The DECIDE-AI checklist was published in 2022 to assist the reporting of the growing number of AI based clinical DSS to ensure safety and evaluate the human factors surrounding the use of such systems. The guideline was developed under a consensus based agreement by a multi-stakeholder group. As the publication was produced in 2022, many authors would be unaware of its presence, which could account for the low adherence, particularly in papers published before 2022.

Of the generic reporting items (maximum = 10), only VI (patient involvement) showed low adherence, while the AI specific reporting items (maximum = 28) 3c (steps to familiarise user), 6a (identifying malfunctions), 6b (how patient risk was delt with), 7 (human factors), 10a (user exposure), 10b (changes to clinical work flow), 13a (safety and error), 14a (usability evaluation) and 14b (user learning curves) were not reported at all. These reporting items predominately represent participation, safety, and human factors, all key elements of a decision support system. Without usability, a DSS could easily become unused or cause adverse events owing to the user not being able to easily understand and use the system.

Despite the low adherence to the DECIDE-AI guidance, it would be prudent to reassess compliance in the future to determine if adherence to this novel reporting guideline has increased as authors become aware of its presence. By abiding by these minimum reporting standards authors can systematically report AI driven DSS consistently and duly consider the “proof of clinical utility at small scale, safety, human factors evaluation and preparation for larger scale summative trials” [8]. It would also be useful for other disciplines of medicine to repeat this study to see how they adhere to the DECIDE-AI checklist, allowing for comparison and knowledge transfer between specialities. Although different specialities have their own technical needs, such knowledge transfer could assist with usability and integration of systems into current workflows to optimise patient care and outcomes.

This study has several strengths presenting the adherence to a novel guideline designed by a consensus process. Adherence to these guidelines standardised the reporting and allows for comparability, an important factor with the rising number of papers citing AI to aid healthcare. The presented review assesses a narrow time-frame of papers that represent the current literature of AI in glaucoma care and the exponential growth of AI applications in medicine, thus providing a timely narrative of the importance of standardised reporting when building the evidence base for including AI DSS into the current workflow of healthcare systems.

Despite the many strengths of this study we also acknowledge some limitations, such as the use of informed consent as a proxy for early clinical evaluation. As noted within the DECIDE-AI explanation and elaboration, the stage specific terminology is ambiguous. Therefore, the authors felt that use of informed consent would be a good discriminator between in silico and live evaluations of this novel technology. Another factor to consider is the novelty of both the body of literature surrounding AI applications in glaucoma care and the checklist, therefore it would be interesting to review in the future if there is a significant difference in the reporting of early clinical evaluation of these DSS in glaucoma care owing to the publication of the DECIDE-AI guideline.

This systematic review highlights the current under-use of the DECIDE-AI checklist when authors are reporting the early stage clinical evaluation of DSS driven by AI for identifying glaucoma or its progression. Generally, authors adhered well to the generic reporting items, while falling short on the AI specific reporting items. In particular, this review found that authors underreported the human factors and those of patient and public involvement associated with the reporting guideline. As the DECIDE-AI guidance was only published in 2022, it is hoped that journal editors and authors will soon adopt citing it in their work to help improve the standardisation of reporting and robustness of this specific stage of the evaluation of AI driven DSS to allow systematic comparisons between model evaluations.

## Supplementary information

Supplemental Figure 1

Supplemental Figure 2

Supplemental Figure 3

## Data Availability

NA
