# Supplementary material for: AI for glaucoma, Are we reporting well? a systematic literature review of DECIDE-AI checklist adherence
Source: Eye (Lond). 2025 Feb 18;39(6):1070–80. doi: 10.1038/s41433-025-03678-5 (PMC11978933; doi:10.1038/s41433-025-03678-5)
Supplement: Supplementary file 1 — Supplemental Figure 1 [file 41433_2025_3678_MOESM1_ESM.pdf]

Supplemental Figure 1, Search Strategy

1	artificial intelligence.mp. or exp artificial intelligence/
2	deep learning.mp. or exp deep learning/
3	machine learning.mp. or exp machine learning/
4	1 or 2 or 3
5	exp glaucoma/ or glaucoma.mp.
6	exp diagnosis/ or diagnos*.mp.
7	progress*.mp.
8	6 or 7
9	4 and 5 and 8
