# Supplementary figures and images for: AI for glaucoma, Are we reporting well? a systematic literature review of DECIDE-AI checklist adherence
Source: Eye (Lond). 2025 Feb 18;39(6):1070–80. doi: 10.1038/s41433-025-03678-5 (PMC11978933; doi:10.1038/s41433-025-03678-5)

Supplemental Figure 2, Distribution of AI Method used

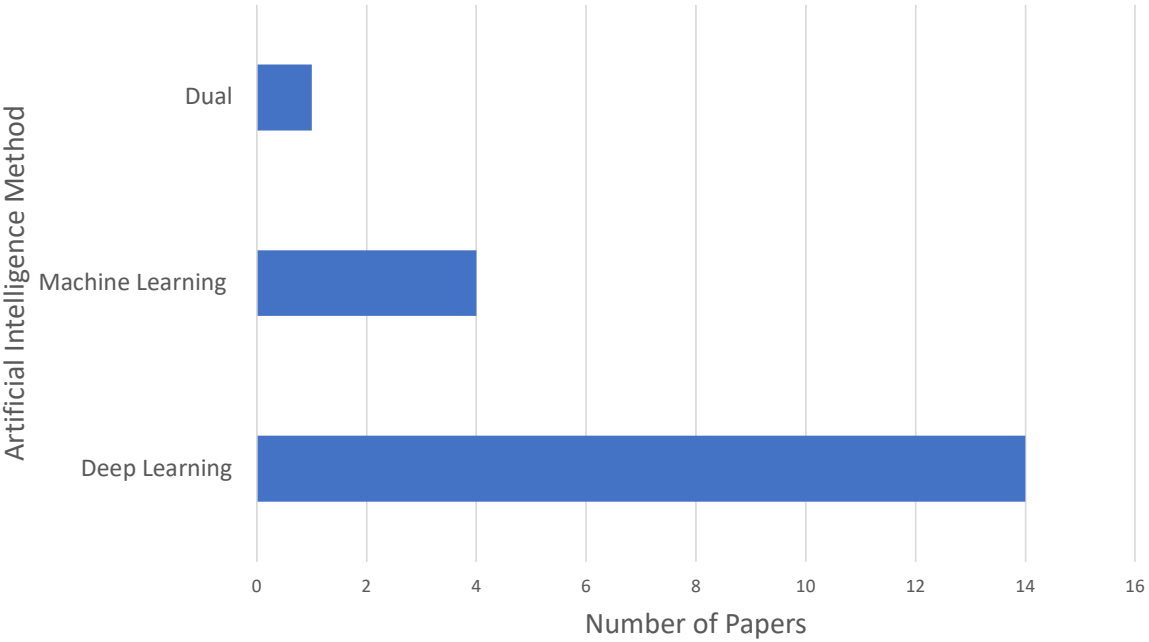

Supplement: Supplementary file 2 — Supplemental Figure 2 [file 41433_2025_3678_MOESM2_ESM.pdf]

Supplemental Figure 3, Distribution of Data inputs

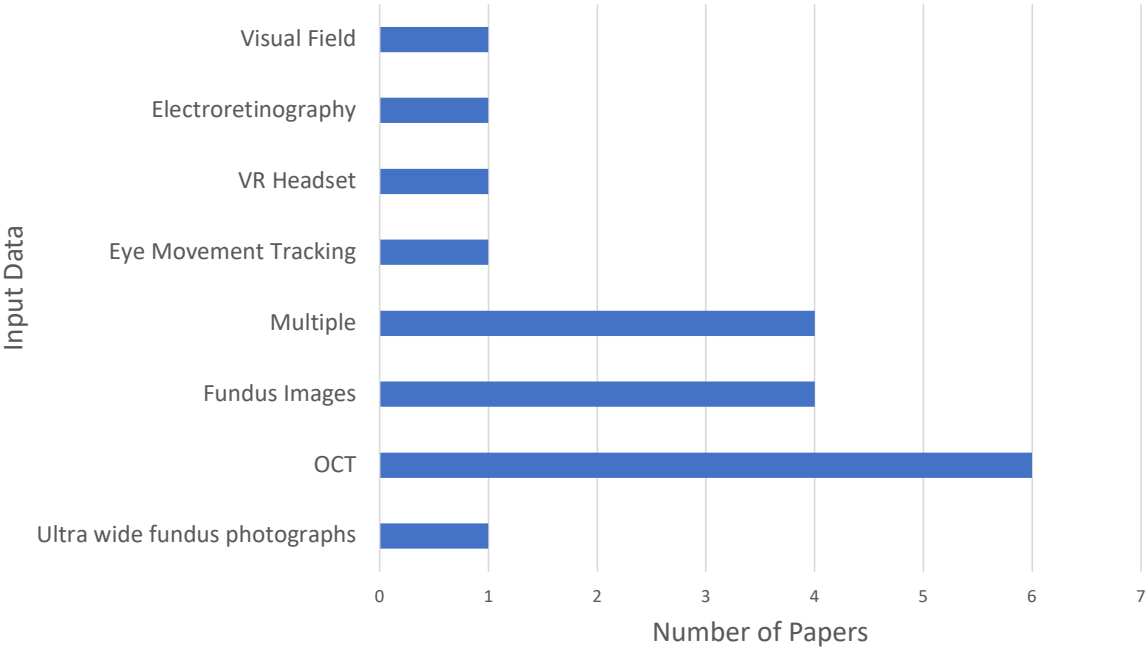

Supplement: Supplementary file 3 — Supplemental Figure 3 [file 41433_2025_3678_MOESM3_ESM.pdf]
